# Model Predictive Control with Optimal Modelling for Pneumatic Artificial Muscle in Rehabilitation Robotics: Confirmation of Validity Though Preliminary Testing

**DOI:** 10.3390/biomimetics10040208

**Published:** 2025-03-28

**Authors:** Dexter Felix Brown, Sheng Quan Xie

**Affiliations:** School of Electronic and Electrical Engineering, University of Leeds, Leeds LS2 9JT, UK; el18dfb@leeds.ac.uk

**Keywords:** pneumatic artificial muscle, control systems, machine intelligence, rehabilitation robotics

## Abstract

This paper presents a model predictive controller (MPC) based on dynamic models generated using the Particle Swarm Optimisation method for accurate motion control of a pneumatic artificial muscle (PAM) for application in rehabilitation robotics. The physical compliance and lightweight nature of PAMs make them desirable for use in the field but also introduce nonlinear dynamic properties which are difficult to accurately model and control. As well as the MPC, three other control systems were examined for a comparative study: a particle-swarm optimised proportional-integral-derivative controller (PSO-PID), an iterative learning controller (ILC), and classical PID control. A series of different waveforms were used as setpoints for each controller, including addition of external loading and simulated disturbance, for a system consisting of a single PAM. Based on the displacement error measured for each experiment, the PID controller performed worst with the largest error values and an issue with oscillating about the setpoint. PSO-PID performed better but still poorly compared with the other intelligent controllers, as well as still exhibiting oscillation, which is undesirable in any human–robot interaction as it can heavily impact the comfort and safety of the system. ILC performed well with rapid convergence to steady-state and low-error values, as well as mitigation of loads and disturbance; however, it performed poorly under changing frequency of input. MPC generally performed the best of the controllers tested here, with the lowest error values and a rapid response to changes in setpoint, as well as no required learning period due to the predictive algorithm.

## 1. Introduction

Physical therapy for the restoration of motion in physically and neurologically injured limbs is an incredibly important procedure for the millions of people suffering limited motion worldwide, but with the increasingly limited healthcare services available this puts incredible strain on therapists and healthcare workers. According to the World Stroke Organization, between the years 1990 and 2019 there were more than 12.2 million new stroke cases each year, and by 2019 over 101 million people lived post-stroke [1]. With 20–25% of stroke survivors never being able to walk without assistance, and 65% unable to use their affected hands normally [2], this issue is widespread and affects a great number of people every year. In an attempt to reduce the strain on healthcare services and provide the needed care to patients, rehabilitation robotics is an open research field aiming to develop and produce robots capable of giving patients similar treatment to trained therapists. However, the limited functionality of these robots and their high cost have reduced their availability, and the field is still in its infancy. To advance the field, this paper presents research into the actuators used in rehabilitation robots and potential control systems which may improve their functionality.

The type of actuator used in a rehabilitation robot is important for several reasons. They must be capable of repeated, accurate motion to provide the treatment necessary for patients requiring rehabilitation and be able to apply sufficient torque to the patient to move their limb along the required motion, or to resist their motion in the case of active resist exercises but be controllable such that the torque output does not exceed safe values. Specifics of actuator design and control for rehabilitation robotics are detailed in [3]. Another desirable factor is intrinsic compliance, which can help increase safety and comfort, allow users a degree of freedom of motion, as well as increase the transparency of the system by reducing patient–machine interaction forces. Several compliant actuators exist which are popular in the field of rehabilitation robotics, including Series Elastic Actuators (SEAs) and Pneumatic Artificial Muscles (PAMs), but controlling these actuators comes with its own problems. Due to the nonlinear nature of intrinsically compliant actuators, they act dynamically to input rather than linearly. This makes more traditional controllers like PID less effective in providing accurate motion. Reviews of soft and compliant actuators in rehabilitation robotics are presented in [4,5].

The use of pneumatic actuators in lower-limb rehabilitation robots has grown over the past few years, as explored in the review presented in [6]. Various pneumatic actuators are popular in the field for their compliant dynamics and interactive nature, including McKibben artificial muscles, rubbertuators, air muscles, pneumatic artificial muscles, and pneumatic muscle actuators. It is also mentioned in this review that while PID controllers are suitable for controlling pneumatic actuators, the nonlinear dynamics and complexity of the actuators may not be accurately controlled without further integrated control paradigms. Among those mentioned are various adaptive controllers using PID, a neuro-fuzzy network controller, neural-network based control and sliding mode control. One final suggestion states that a more complicated controller may not be the best approach to this problem, as additional sensors and impedances would increase the overall complexity of the system and make it more difficult to use, and a simpler controller is preferred.

This sort of compliant actuator is used for various purposes in the field, and in different configurations. The robot presented in [7] uses an antagonistic pair of pneumatic muscles to control a finger rehabilitation robot. This system also uses a magnetorheological dampener, and in experiments both open- and closed-loop PID control systems are implemented. A powered ankle–foot orthosis (PAFO) using a pair of PAMs to provide two degrees of freedom is presented in [8]. This system uses a sliding-mode controller for actuator control. The system presented in [9] consists of three separate pneumatic subsystems for body weight support, postural support, and gait orthosis. This system is a full-body gait rehabilitation system and uses an Interval Type-2 Fuzzy Sliding Pulse-Width Modulation Control to allow for proper control of the nonlinear pneumatic systems. A control system is developed in [10] which incorporates the RBF neural network and sliding mode control to ensure adaptive and disturbance-resistant control of a single-degree-of-freedom rehabilitation robot using a pair of antagonistic PAMs. The robot is designed to function for multiple different limbs. A neural network-based controller is also used in [11] for a hand rehabilitation robot utilising flexible pneumatic muscles. The intelligent controller has adaptive elements allowing it to accurately control the position of the orthosis despite interference forces and the nonlinearity of the actuators. Another control scheme designed to accurately control pneumatics is presented in [12] with a multiple-input single-output model free-adaptive control scheme. This controller is applied to a single DOF multi-joint rehabilitation robot which uses two PAMs similar to [8]. Experiments presented in [13] aim to determine the dynamic characteristics of PAMs with the intention of accurate servo control. Nonlinear pressure flow values are compensated for using a lookup table, and position control is achieved using a model-based PID controller. This simple control structure achieved good closed-loop regulation and position tracking.

As shown in the literature, there is sufficient interest in pneumatic actuators in the field of rehabilitation robotics to make it an area of growing research, particularly the problem of accurate control. Accurate, reliable, and flexible motion tracking in a rehabilitation robot is essential for its effective use in a real rehabilitation setting. As professional therapists set the desired exercise motions, it is important to have them be properly followed to ensure proper rehabilitation outcomes for patients, as well as ensure their safety in using a robot. A control system able to perform well under many different input requirements also makes the robot more flexible in the conditions and stages of recovery it can treat. However, while good control performance is shown in many studies, there is a lack in comparison data between different intelligent control approaches in PAM-actuated rehabilitation robotics.

To address this limitation and investigate a novel application of prediction and optimisation algorithms, this paper presents preliminary implementation and testing of a Model Predictive Control (MPC) scheme utilising a Particle Swarm Optimisation-based (PSO) modelling method to generate dynamic models of the actuator, tested experimentally on a single-PAM setup in a simple hanging position as a feasibility study for eventual application of the controller to a PAM-driven rehabilitation robot. The development and experimental validation of the PSO-based modelling method is presented in [14]. This approach involves a phenomenological approximation of the PAM to a mass-spring-damper system, optimising the three parameters using a PSO algorithm in a three-dimensional search space. Based on preliminary testing, the linear model generation method, in which a single model is used to approximate the PAM’s motion over a specific pressure input range, will be used here. The switching issue present in the piecewise and inflate–deflate modes has a magnified effect when used in conjunction with a control algorithm; as such, the linear method was considered the best approach for motion accuracy.

In order that this controller is properly validated in comparison with other controllers commonplace in the field, two other intelligent control strategies were applied to the system, as well as one classical control strategy: iterative learning control (ILC), Particle Swarm Optimised Proportional Integral Derivative Control (PSO-PID), and classical PID control. While PID control is generally regarded as unsuitable for use with nonlinear systems based on its linear nature and poor approximation of time-variant systems, its use is prevalent in control theory as a computationally and mathematically efficient algorithm. As such it will be included in this study as a comparison point to demonstrate the improved effectiveness of intelligent control on the PAM system.

MPC uses a dynamic model of the system to predict over a finite period the behaviour of the system and calculates the control output required to best optimise some cost function over that period. Using this predictive method, the time-variant characteristics of the system are accounted for, and nonlinearities can be included in the model of the system. This makes MPC a suitable controller for PAMs. However, the need for a model of the system makes it difficult to apply to complex systems and actuators. This is especially true for systems including uncertainties and multiple nonlinear factors such as PAMs, as models for such systems tend to include multiple assumptions and simplifications, giving model-based controllers an inherent trade-off between accuracy and computational efficiency. In [15], a switching model predictive controller is developed for a PAM for the purpose of testing a novel modelling method using PieceWise Affine model approximation. The PAM is initially estimated as a spring-damper system with a second-order differential equation of motion, but in the piecewise modelling estimation the second-order element is ignored. A single PAM is set up vertically to measure displacement with no load attached. The controller, in comparison with the PID control of the same system, is found to have lower motion error values and faster convergence. A 2 DoF robot actuated by two antagonistic pairs of PAMs as presented in [16] which uses MPC for motion control. Simulations show that the controller provides accurate motion control with small errors and stable steady-state behaviour, with the model used for the system dynamics being a nonlinear eighth-order set of differential equations, with polynomial functions serving as the characteristics of the PAMs. A discrete-valued model-predictive controller dubbed DVMPC is presented in [17], and improved upon in [18], for motion control of a novel hybrid-pneumatic electric actuator and a double-acting pneumatic cylinder, respectively. Results from both studies show that novel controllers outperform the state-of-the-art sliding mode control by a significant margin. A lower limb orthosis powered by PAMs and controlled by a neural network-based nonlinear MPC is presented in [19]. In online experiments, the system meets requirements for each different participant, and the major concerns for inhibited system performance are the PAM’s intrinsically slow rate of motion owing to the maximum allowed air flow into and out of it, and human–machine interaction sensing. MPC has been proven suitable for accurate motion control of PAMs; however, the modelling required for this control method produces another level of complexity, as this in itself is a separate area of study.

ILC is popular for use with nonlinear actuators as it does not rely on a complex model of the system, rather measuring the desired and actual outputs and iteratively calculating the best control signal based on the difference. This makes it suitable for time-varying systems, and there are many examples of its use in rehabilitation robotics using PAMs. The system RUPERT presented in [20] uses ILC for assistive reach-to-grasp tasks actuated by a series of PAMs, where its use is attributed to the need for a suitable controller for a nonlinear and time-varying system, as well as the repetitive nature of attempting the same task multiple times. Another rehabilitation/assistive robot utilising PAMs is presented in [21], in which uses an improved PID controller has issue with phase lag relieved using ILC to iteratively adjust control parameters, fixing this lag issue and reducing output error. A simulation of a PAM presented in [22], utilising ILC to account for the nonlinear and time-varying nature of the actuator, shows results proving the algorithm is capable of accounting for these characteristics, as well as uncertainties in the system. Based on the widespread use and research into ILC in the field of rehabilitation robotics and specifically its usefulness in controlling PAMs, it was chosen as a comparison for the developed MPC in this study.

Similarly to learning algorithms, optimisation is often used to improve the performance of controllers for complex systems, with PSO being a popular optimisation algorithm. PID is often the target of such optimisation as its functionality is heavily tied to how well the small number of parameters are tuned to the system it is to control. An upper limb exoskeleton robot using this method of optimised control is presented in [23] alongside a method using an improved version of classical PSO, Artificial Bee Colony Optimisation. A lower limb rehabilitation robot presented in [24] also uses improved PSO-PID control. Both these studies found their optimised controllers to be robust and have more accurate motion control. The study presented in [25] develops a dynamic neural network and PID controller for control of PAMs, the parameters of which are optimised using the PSO algorithm with Integral Time Absolute Error as the objective function. Another study using a similar technique is presented in [26] with adaptive and classical synergetic controllers developed for a robotic arm actuated by a PAM.

The experiments presented in this paper aim to test and compare the developed MPC with classical ILC, PID, and PSO-PID in controlling the actuation of a single PAM in online control experiments, for future implementation to a complete PAM-drive rehabilitation robot. The main results for determining effectiveness will be motion tracking accuracy, response to disturbance, and smoothness of motion, as these are necessary for the controller to function well as part of a rehabilitation robot utilising PAMs for actuation. The experiments will take place on a single PAM set up to accurately measure displacement. Weights will be used to apply resistive force to the actuator, as well as to simulate disturbances to the system. Each controller will be implemented and tested with the same test scheme, and the displacement error values will be measured and analysed to determine how well they perform comparatively.

The contributions of this study include development and implementation of a model predictive controller for accurate PAM motion using the PSO-based modelling method presented in [14] to generate dynamic models of the system, experimental data for comparison of the performance of the MPC as well as other popular controllers in the field of rehabilitation robotics—ILC, PID, and PSO-PID—using an experiment plan designed to simulate the motion and load requirements of PAMs in a rehabilitation robot, as well as a comparative discussion comparing the relative strengths and weaknesses of each controller. Based on the limited examples of comparative studies, this experiment including multiple commonplace controllers will help in quantifiably demonstrating the relative benefits and shortcomings of these controllers and help fill this gap in the literature. The overall aim of this research is to determine the feasibility of the developed MPC, as well as classical ILC and optimised PID controller, for accurate motion control of PAMs in a rehabilitation robot setting, with the future aim to implement the developed algorithms in a PAM-driven rehabilitation robot.

## 2. Materials and Methods

### 2.1. Controllers

#### 2.1.1. Model Predictive Control (MPC)

**Figure 1:**
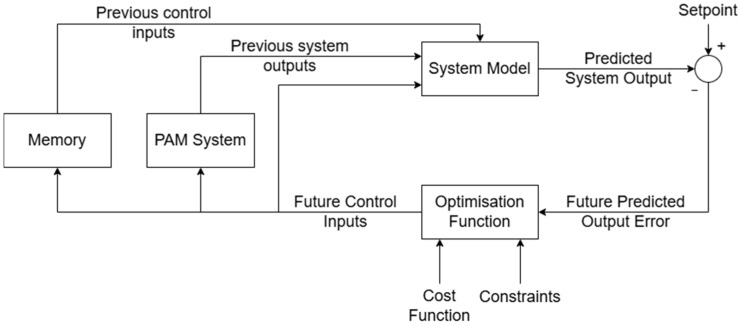
MPC structure. The system model is generated according to the methods described in [14].

MPC uses a model of the system as well as prior knowledge of the input sequence to predict the movement of the actuator in advance for a given future time period. This allows for accurate control of nonlinear systems for any input sequence and is therefore usable for various applications. MPC also has the option of constraining many aspects of the control sequence, including minimum and maximum control values for both the control output and the predicted control output, as well as minimum and maximum values for the rate of change of control values. However, the necessity of a mathematical model of the system can cause issues in development, especially for a complex system such as a PAM, as these models often include simplifications and assumptions resulting in inaccuracies. The controller also requires that the entire input sequence be known beforehand, and the predictive nature prevents it from being used in modes other than passive movement, such as resistive training in rehabilitation. The structure of the MPC algorithm is shown in Figure 1.

At each sampling period t the state of the system is taken and via an optimization function, a cost-minimising control output is calculated for a time horizon defined by the prediction period T. Once a cost-minimal control trajectory starting at the sampled state and for the time period [t,(t+T)] is found, the first step of this strategy is applied to the system. The remainder of the calculated control trajectory is discarded, and the process is repeated from the new system state. While MPC is an optimal control strategy as it uses a cost optimisation method for control, the process by which it calculates this control is not optimal as a great many calculations are discarded.

The optimisation algorithm used here is linear quadratic programming, the cost function of which is described by Equation (1):

(1) $$J=\sum_{t}^{T}q{\left(\hat{y}-s\right)}^{2}+\sum_{t}^{T}r{∆u}^{2}$$

where t is the current time step, T is the prediction period, q is the output error weight parameter, $\hat{y}$ is the predicted system output, s is the setpoint, r is the control weight parameter, and ∆u is the predicted change in the control value such that $∆u=u_{k}-u_{k-1}$ with $u_{k}$ being the control value taken at time step k. For this MPC implementation, the output error weight was set to 1 and the control weight was set to 20.

Based on this cost function, the optimal control is achieved by solving Equation (2) for u:

(2) $$\frac{\delta J}{\delta u}=0$$

As MPC relies heavily on the accuracy of the model of the system to properly optimise potential control solutions, it is important the model used approximates the PAM as closely as possible. As discussed in [14], modelling PAMs is particularly challenging due to their dynamic behaviour. The PSO-based modelling method used there will be employed here to generate mathematical models for the MPC based on the proposed mass-spring-damper approximation. To get a more accurate model response without the undesirable jumping from a piecewise model, a single linear phenomenological model will be generated for each required motion, accounting for the position- and time-variant behaviour of the PAM over different stroke lengths and pressure input ranges.

#### 2.1.2. Iterative Learning Control (ILC)

The ILC improves tracking performance over multiple iterations of the same input sequence, thereby progressively reducing errors. Using this control algorithm, long-term stability and performance is improved; however, the initial learning period is generally unstable and inaccurate. Therefore, reducing this initial period by speeding up the learning rate can improve the functionality of the controller. This control method is model-free, as the controller output is calculated using error feedback. This is a desirable trait for controllers of nonlinear systems as it removes the need for a complex mathematical model, which would likely include simplifications and assumptions.

The standard equation for control output for ILC is shown in Equation (3):

(3) $$u\left(t\right)=u_{old\;}\left(t\right)+pe_{old}(t)$$

where u(t) is the new control signal, $u_{old}(t)$ is the control signal at the same time step within the previous learning period, $e_{old}(t)$ is the error at the same time step within the previous learning period, and p is the learning rate parameter which is manually tuned.

#### 2.1.3. Particle Swarm Optimised PID Control (PSO-PID)

PID control is generally regarded as unsuitable for nonlinear systems such as PAMs as it treats the input–output relationship of the system as position- and time-invariant. Introducing PSO algorithms to optimise the parameters of the PID controller can both improve this shortcoming by calculating optimal parameters based on the behaviour of the PAM over the required motion. The optimisation algorithm is run offline to generate parameters for the PID controller for online experiments.

The equation for velocity update of each particle for classical PSO is shown in Equation (4):

(4) $$V_{i}\left(k+1\right)=WV_{i}\left(k\right)+C_{1}Rand\left(X_{i}best\left(k\right)-X_{i}\left(k\right)\right)+C_{2}Rand(Gbest\left(k\right)-X_{i}\left(k\right))$$

The equation for position update is shown in Equation (5):

(5) $$X_{i}\left(k+1\right)=X_{1}\left(k\right)+V_{i}(k+1)$$

where i is the current particle; k is the current iteration of the algorithm; V is the particle’s velocity in the search space; X is a particle’s position in the search space quantified by its parameters; Rand is a random value between 0 and 1; $X_{i}best(k)$ is the position of the current particle which achieved the best (lowest) fitness; Gbest(k) is the global best position of all particles in the swarm; and W, $C_{1}$, and $C_{2}$ are algorithm parameters that are tuned manually.

To use PSO to optimise parameters for PID control, a 3-dimensional search space is used, optimising the parameters $K_{c}$, $T_{i}$, and $T_{d}$. To converge on an optimal solution, the objective function used for swarm fitness is the RMSE of motion tracking over a single input wavelength.

### 2.2. Experimental Setup

**Figure 2:**
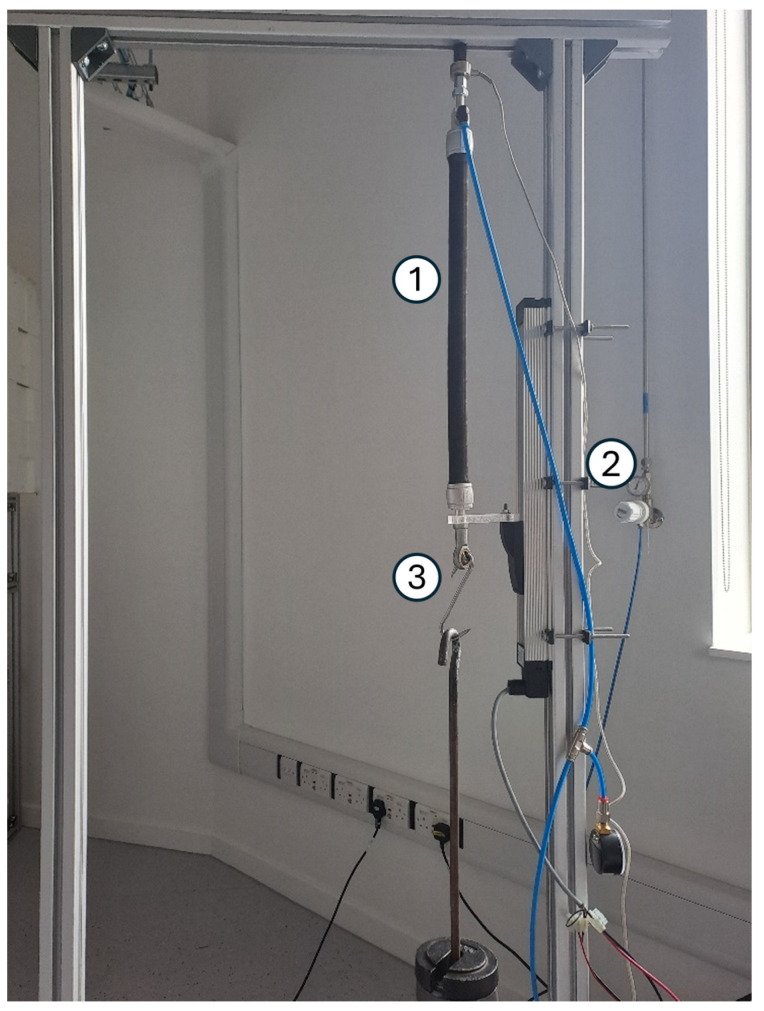
The experimental setup, showing the FESTO DMSP-20-400N PAM (1), FESTO MLO-POT-300-TLF displacement encoder (2), and external load attachment (3).

The system designed for testing the developed controllers includes a single PAM setup in a vertical position, with the fixed end connected to a frame and the unaffixed end connected to a linear encoder as the output measure of these experiments is the actuator’s displacement. Pressure input and output to the PAM is controlled by a proportional pressure regulator, the input voltage of which is the controlled variable of the system. Figure 2 shows a picture of the PAM setup with the encoder sensor and external weight attachment.

The actuator to be used in these experiments is a FESTO DMSP-20-400N PAM. The model of the linear encoder is a FESTO MLO-POT-300-TLF with a resolution of 0.01 mm and stroke length of 300 mm, both of which are sufficient for the system. The proportional pressure regulator used for controlling pressure input to the PAM is a FESTO VPPM-6L-L-1-G18-0L6H. FESTO is based in Esslingen am Neckar, Germany. The system is held in place with a frame consisting of slotted aluminium extrusion and the PAM is positioned vertically to ensure motion and forces are not adversely affected by the actuator’s intrinsic compliance regarding gravity.

To control the system, a National Instruments RoboRIO is connected to a desktop PC and coded using National Instruments LabVIEW 2016. National Instruments is headquartered in Austin, Texas, United States. The output of the displacement encoder is input to the RoboRIO to be measured on the display.

**Figure 3:**
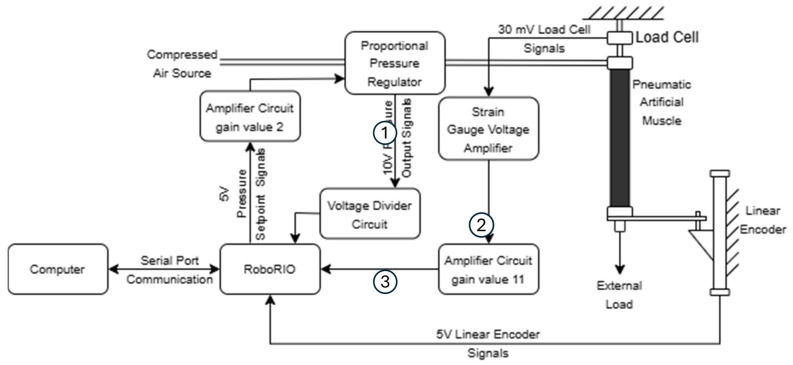
A diagram of the experimental setup, circuitry, and computer communications.

Figure 3 shows a diagram of the experimental setup, including communication with the embedded controller and computer.

### 2.3. Experiment Plan

Each experiment will involve a specific motion as input to each controller, along with the addition of external weights applied to the PAM, and adding/removing them at certain points during motion. Table 1 shows the experiment outline as well as the main purpose of each set of experiments. Each experiment, including input motion setpoints and weights used, will be performed unaltered for each controller to ensure the results are comparable.

### 2.4. Algorithm Parameters

#### 2.4.1. MPC Model Generation

**Figure 4:**
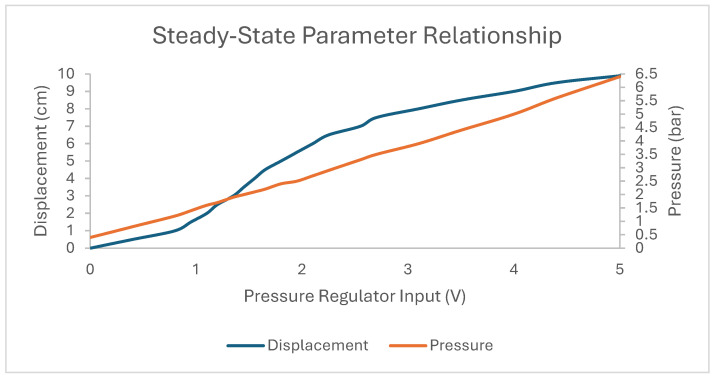
Steady-state relationships between voltage input to the pressure regulator, internal air pressure in the PAM, and actuator displacement.

The PSO-based modelling method presented in [14] is used here to generate dynamic models of the PAM for use in the MPC algorithm. A PSO algorithm with a swarm size of 50, W=0.9, $C_{1}=C_{2}=2$, and run for 20 iterations was used to generate each model using the method described in [14]. The fitness value was calculated using the actual PAM motion with a sin wave voltage input to the pressure regulator, the amplitude of which was based on the steady-state displacement of the PAM at certain voltage inputs, as shown in Figure 4, such that the required displacement upper and lower bounds for the comparative experiment were reached during model generation. Using this method, a set of model parameters were generated which best described the PAM’s behaviour for each required motion in the experiment plan, allowing for more accurate control through the MPC algorithm. The parameters of these models are shown in the MPC model parameters column in Table 2.

#### 2.4.2. PID Parameters

The parameters used for the classical PID controller were $K_{c}=0.6$, $T_{i}=0.01$, and $T_{d}=1e^{-5}$. These parameters were tuned manually.

#### 2.4.3. ILC Parameters

The learning rate used for the ILC in each experiment was p=0.2. This value was tuned manually to optimise rate of convergence to the setpoint while minimising overfitting.

#### 2.4.4. PSO-PID Parameters

To improve on the classical PID algorithm, PSO was used to generate optimal parameters for the three control values. Based on the dynamic behaviour of the PAM across its stroke length, as well as keeping the controller as computationally simple as possible while maintaining accurate motion tracking, a set of parameters, each of which included values $K_{c}$, $T_{i}$, and $T_{d}$, was generated for each motion required by the comparative experiment plan. This allowed for the most optimal PID control available based on the algorithm for each experiment.

Each set of parameters was generated using a PSO algorithm with a swarm size of 10, W=0.9, $C_{1}=C_{2}=2$, and run for 10 iterations. The position values of each particle corresponded to the $K_{c}$, $T_{i}$, and $T_{d}$ parameters of the PID controller. The fitness of each particle was calculated as the RMSE between the desired setpoint motion of the PAM displacement and the actual PAM displacement under PID control, using that particle’s parameter values, over two wavelengths of motion. The resulting parameter sets are shown in the PSO-PID parameters column in Table 2.

## 3. Results

**Figure 5:**
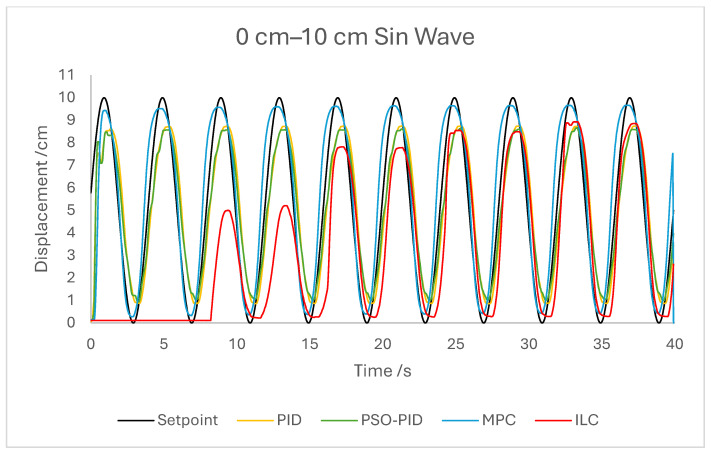
Graph showing the setpoint waveform and PAM displacement under each control scheme during the 0 cm–10 cm sin wave experiment.

**Figure 6:**
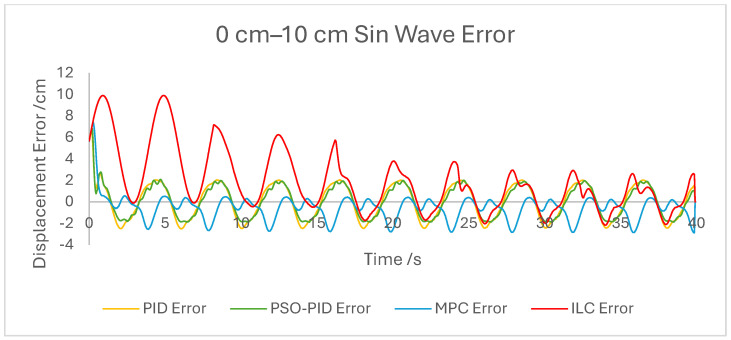
Graph showing displacement error of the PAM under each control scheme during the 0 cm–10 cm sin wave experiment.

**Figure 7:**
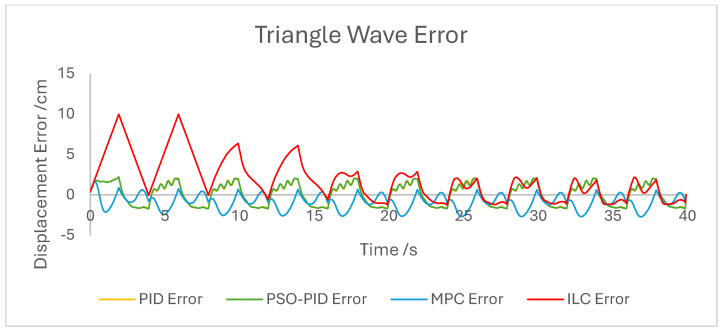
Graph showing displacement error of the PAM under each control scheme during the triangle wave experiment.

**Figure 8:**
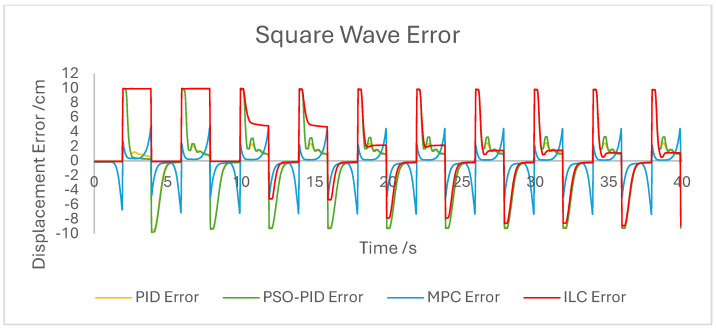
Graph showing displacement error of the PAM under each control scheme during the square wave experiment.

**Figure 9:**
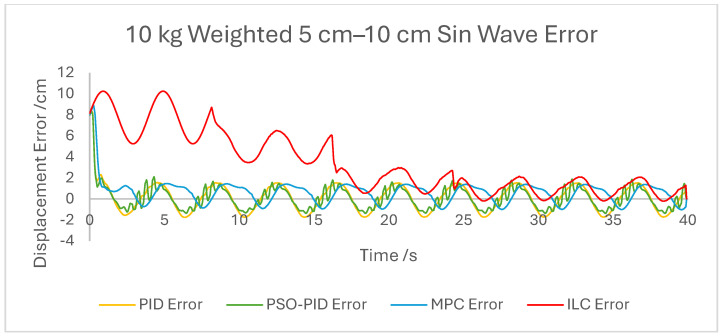
Graph showing displacement error of the PAM under each control scheme during the 10 kg-weighted 5 cm–10 cm sin wave experiment.

**Figure 10:**
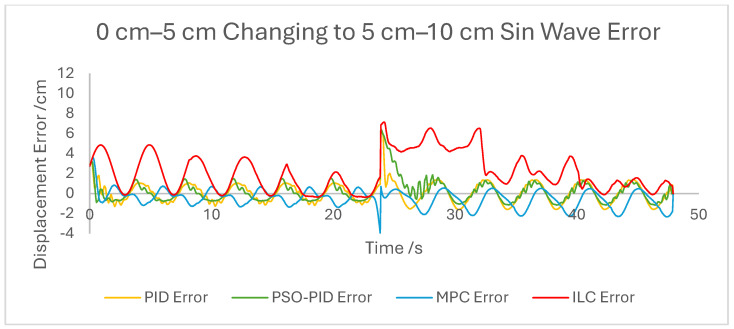
Graph showing displacement error of the PAM under each control scheme during the 0 cm–5 cm changing to 5 cm–10 cm sin wave experiment.

**Figure 11:**
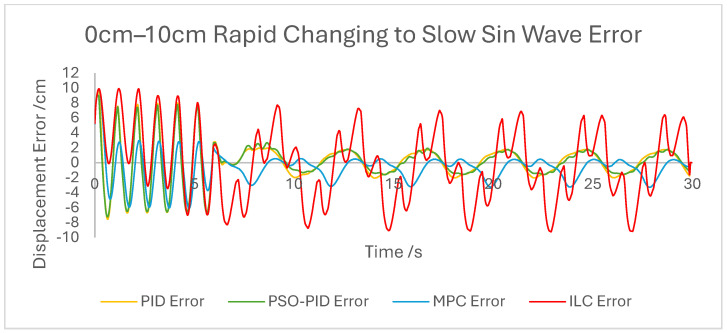
Graph showing displacement error of the PAM under each control scheme during the rapid changing to slow motion 0 cm–10 cm sin wave experiment.

**Figure 12:**
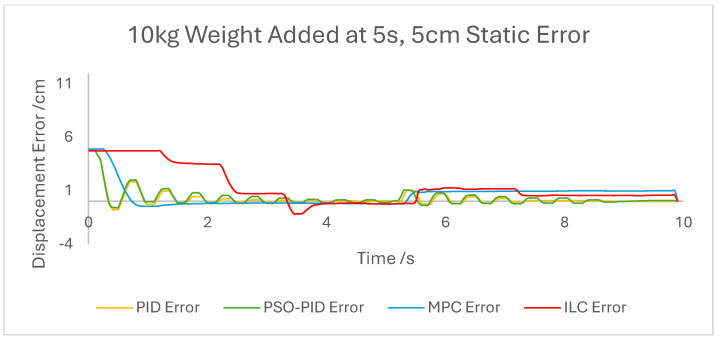
Graph showing displacement error of the PAM under each control scheme during the 10 kg added weight experiment.

The displacement of the PAM under each control scheme was measured for each experiment and overlaid for comparison, as shown in Figure 5. The displacement error for each experiment was also calculated and overlaid for each experiment. Figure 6, Figure 7, Figure 8, Figure 9, Figure 10, Figure 11 and Figure 12 show this error results from some of the experiments best demonstrating the relative performance of the controllers.

## 4. Discussion

The dynamic properties of the PAM heavily impact the accuracy of motion under all control schemes at a high-velocity input, such as during the rapid sin wave experiments, in which a 1 Hz setpoint waveform is used rather than the 0.25 Hz used in the other experiments, as well as the square wave experiments. Rather than a fault in the controllers, this is mostly a reflection of the maximum change in displacement the actuator is capable of achieving. This also has an effect under a triangle wave setpoint, as constant velocity is difficult to achieve with dynamic input/output behaviour. The higher frequency experiment, while not an accurate representation of actuator requirements in a rehabilitation setting, was performed to test the higher limits of speed response for the PAM and controllers. The error values for these experiments were generally the highest; however, the comparison between the controller is useful as will be discussed.

The performance of the PID and PSO-PID controllers were generally consistent with each other. Both exhibited oscillating behaviour during most motions, including during constant displacement experiments and during the stationary points of the square wave setpoints. This was exacerbated with the addition of external loads, especially noticeable in the static experiments where the oscillation was mostly present before the weights were removed and after they were added. Both PID control schemes had a poor response time under square wave setpoints compared to the other controllers, with noticeably larger errors at the changing points in the waveform as well as slower settling time; however, the steady-state error at the static points was generally small. The poor response time can also be seen in the sin wave experiments with the PAM motion lagging behind the setpoint. The PSO-PID controller showed lower error values in all experiments than classical PID, and the oscillating behaviour was less pronounced, except during the upper stroke length of the PAM and during the triangle wave setpoint. This behaviour is likely due to a closer fitting to the setpoint at points where the nonlinear nature of the PAM is most pronounced, causing the controller to quickly switch between increasing and decreasing pressure input in an attempt to meet the desired displacement.

The behaviour of the ILC was as expected in all experiments in terms of learning, with convergence to a steady state reached after eight wavelengths, or four iterations of the learning algorithm. Once a steady state is reached the error is typically similar in magnitude to that of the PSO-PID controller, but with no evidence of oscillation about the setpoint. Motion under the ILC is generally smoother than under PID control. ILC suffers from similar response-time issues to PID control, with the PAM motion lagging behind the setpoint even after a steady state is reached. The response time during square wave motion is slightly better than that of PID, with lower settling time and notably no oscillation at the static points, but instantaneous errors are similar in magnitude at the changing points of the waveform. Under external load the ILC performs well, with little impact on the learning rate or the overall motion accuracy. Addition and removal of weights during motion and during static displacement is accounted for by the learning algorithm; however, with a constant setpoint the steady state error under ILC is the worst of the controllers tested here. Under changing setpoints the ILC can respond to the change in a similar time to the initial learning period, but this does result in worse overall error. The learning algorithm does not respond properly to changes in the frequency of the setpoint during motion, with the initial frequency maintained throughout without changing to match the setpoint. This is due to the learning period being based on the wavelength of the setpoint. A more sophisticated learning algorithm could improve on this with a better-defined learning period.

Overall, based on average error values across the experiments’ runtimes as well as maximum error magnitudes, the motion of the PAM under MPC is the most accurate. The response time issue present in the other controllers tested here is removed by the predictive algorithm, although in some cases the actual motion precedes the setpoint, which is a unique issue. With square wave input this is most noticeable, as the PAM motion under MPC changes direction before the changing points of the waveform, resulting in overall lower error values. Under most setpoints the error is lowest under MPC with smoother motion than PID and, as to be expected, a faster settling time than ILC. One major downside of this implementation of MPC is that the model used does not consider external loads to the system, and as such the controller does not change input based on additional loads to the PAM, resulting in larger error values when weights are added. Under rapid motion the predictive algorithm works somewhat against the PAM motion. The system itself is incapable of changing velocity at the rate required by the setpoint, usually resulting in a lagging motion which attempts to reach the peak of the sin wave input. The MPC predicts rapid changes in the setpoint before they occur and changes the direction of motion in advance. While this does result in overall lower error values under MPC than other controllers, the maximum displacement of the PAM is much smaller in practice due to this interaction, which is usually undesirable when displacement accuracy is the priority. The predictive algorithm is capable of adjusting to changes in setpoint waveforms seamlessly. PAM motion immediately before the change in waveform is adjusted to best fit the requirements after the change, so error values are only minorly affected, there is little or no increase in error between the different setpoints, and motion after the change is just as accurate as before.

While the predictive and learning control systems used in this study show promise in accurate motion control for a single actuator, alternative control methodology is required in many rehabilitation robotics applications. Impedance control, such as that presented in [27,28], uses force sensor measurements to determine actuator motion. This feedback system is required in active rehabilitation, in which a user applies force to the robot and the motion is assisted or resisted by the system. This would be impractical for the controllers used here to achieve in their current configuration, so they are not sufficient for every control need of a rehabilitation robot.

## 5. Conclusions

This study presents an intelligent control scheme designed to overcome the nonlinearity of PAMs, Model Predictive Control using PSO-based dynamic modelling, which was implemented on a single PAM setup to test for validity. Three other controllers which are popular in the field of rehabilitation robotics, ILC, PID, and PSO-PID, were also investigated for quantifiable comparison of their performance.

An experiment scheme was designed to test the behaviour of each control scheme under conditions, as would be required in a rehabilitation setting. Displacement of the PAM was measured for each different experiment setpoint and external loading under each of the four control schemes, and the displacement error calculated and compared. Based on these comparisons, it was found that the PID controller performed worst in terms of error magnitude and response time, with undesirable oscillating behaviour in many cases, which was worsened by the addition of weights. Optimised parameters using PSO slightly improved the error values and response times but did not remove the oscillations. The ILC reached a steady state after learning for four iterations in each experiment, with smoother motion and smaller error values than PID. Performance was also not inhibited by external loading, and changes to the setpoint and external loading during motion were accounted for in the next learning iteration. Response time was still slow, however, and the algorithm was incapable of adjusting to changes in frequency. The MPC had the best performance, with smooth motion during all experiments and the overall lowest error values. The prediction algorithm allowed motion in advance of changing setpoints and maintained low error values after changes as well. Due to the method of model generation for the MPC and the lack of feedback, the algorithm was unable to adjust for external loads, and error values were negatively affected by weights.

The results gained in this study prove conclusively that machine intelligence can greatly improve the performance of control systems for PAMs in terms of tracking accuracy, response time, and motion smoothness. Both learning and predictive control methods show excellent performance in most scenarios. Despite being a standard implementation of learning control, the ILC algorithm used in this study showed rapid convergence to accurate motion as well as the ability to sufficiently reject external load and disturbance. The use of additional learning parameters and robust elements could further improve its performance. The predictive nature of MPC especially shows promise as it mitigates many issues which arise in controlling complex actuators like PAMs, including lagging behind the setpoint caused by a limited flow rate of air into the actuator, and the PAM’s tendency to oscillate about setpoint values as it repeatedly attempts to over-adjust. These preliminary results suggest that this implementation of MPC is suitable for further study and future application to a PAM-driven rehabilitation robot. It could also be suitable for other robotics applications, such as in more complex actuator arrangements [29]; however, this is outside the scope of the current research. The overall performance of MPC depends heavily on the accuracy of the model used, and as such a more accurate dynamic model of the PAM would further improve the controller’s performance.

## Figures and Tables

**Table 1:** Experiment Outline.

Experiment	Experiment Purpose	Motion and Weights
Continuous Motion	Determine controller’s motion accuracy with no disturbance for different types of motion	Sin wave, 0 cm–10 cm
		Sin wave, 0 cm–5 cm
		Sin wave, 5 cm–10 cm
		Sin wave, 0 cm–10 cm, rapid motion
		Triangle wave, 0 cm–10 cm
		Square wave, 0 cm–10 cm
Continuous Motion with Weights	Determine the effect of added weight on controller’s motion accuracy	Sin wave, 0 cm–10 cm, 5 kg load
		Sin wave, 0 cm–10 cm, 10 kg load
		Square wave, 0 cm–10 cm, 5 kg load
		Square wave, 0 cm–10 cm, 10 kg load
		Sin wave, 0 cm–10 cm, rapid motion, 10 kg load
		Sin wave, 0 cm–5 cm, 10 kg load
		Sin wave, 5 cm–10 cm, 10 kg load
Continuous Changing Motion	Determine the controller’s response to a change in continuous motion, how quickly they can adapt Changes made after 6 wavelengths	Sin wave, 0 cm–4 cm changed to 0 cm–8 cm
		Sin wave, 0 cm–10 cm changed to 2.5 cm–7.5 cm
		Sin wave, 0 cm–5 cm changed to 5 cm–10 cm
		Sin wave, 0 cm–10 cm, slow motion changed to rapid motion
		Sin wave, 0 cm–10 cm, rapid motion changed to slow motion
Static with Disturbance	Determine controller’s response to disturbance with no noise or motion Performed with static 5 cm setpoint, weights added/removed at 5 s	5 kg load added
		10 kg load added
		5 kg load removed
		10 kg load removed
Continuous Motion with Disturbance	Determine controller’s response to disturbance during motion	Sin wave, 0 cm–10 cm, 10 kg load added after 4 waveforms
		Sin wave, 0 cm–10 cm, 10 kg load removed after 4 waveforms
		Sin wave, 0 cm–10 cm, 5 kg load added and removed after each waveform

**Table 2:** PID control parameters and MPC model parameters.

Waveform	Amplitude (cm)	Offset (cm)	Frequency (Hz)	PSO-PID Parameters			MPC Model Parameters		
				$K_{c}$	$T_{i}$	$T_{d}$	$P_{1}$	$P_{2}$	$P_{3}$
Sinusoidal	10	0	0.25	0	0.801752	0.0191877	8.18 × 10^−6^	0.182535	0.534393
Sinusoidal	5	0	0.25	0	0.34711	0.00266562	0	0.460018	0.191448
Sinusoidal	5	5	0.25	0	1.0466	0.0187886	3.45 × 10^−6^	0.741741	0.012203
Sinusoidal	10	0	1	0	0.801752	0.0191877	8.18 × 10^−6^	0.182535	0.534393
Triangular	10	0	0.25	0	0.570082	0.00962227	9.15 × 10^−6^	0.182535	0.534393
Square	10	0	0.25	0	0.885228	0.00971446	4.96 × 10^−6^	0.182535	0.534393
Sinusoidal	10	0	0.25	5	0.801752	0.0191877	8.18 × 10^−6^	0.182535	0.534393
Sinusoidal	10	0	0.25	10	0.801752	0.0191877	8.18 × 10^−6^	0.182535	0.534393
Square	10	0	0.25	5	0.885228	0.00971446	4.96 × 10^−6^	0.182535	0.534393
Square	10	0	0.25	10	0.885228	0.00971446	4.96 × 10^−6^	0.182535	0.534393
Sinusoidal	10	0	1	10	0.801752	0.0191877	8.18 × 10^−6^	0.182535	0.534393
Sinusoidal	5	0	0.25	10	0.34711	0.00266562	0	0.460018	0.191448
Sinusoidal	5	5	0.25	10	1.0466	0.0187886	3.45 × 10^−6^	0.741741	0.012203
Sinusoidal	4	0	0.25	0	0.442617	0.00526149	5.98 × 10^−6^	0.101338	0.626386
	8	0	0.25	0	0.756974	0.00641648	5.38 × 10^−6^	0.503874	0.154515
Sinusoidal	10	0	0.25	0	0.801752	0.0191877	8.18 × 10^−6^	0.182535	0.534393
	5	2.5	0.25	0	0.626517	0.00597624	7.00 × 10^−6^	−0.10410	0.890396
Sinusoidal	5	0	0.25	0	0.34711	0.00266562	0	0.460018	0.191448
	5	5	0.25	0	1.0466	0.0187886	3.45 × 10^−6^	0.741741	0.012203
Sinusoidal	10	0	0.25	0	0.801752	0.0191877	8.18 × 10^−6^	0.182535	0.534393
	10	0	1	0	0.801752	0.0191877	8.18 × 10^−6^	0.182535	0.534393
Sinusoidal	10	0	1	0	0.801752	0.0191877	8.18 × 10^−6^	0.182535	0.534393
	10	0	0.25	0	0.801752	0.0191877	8.18 × 10^−6^	0.182535	0.534393
Constant	0	5	0	5	0.632498	0.0134572	1.10 × 10^−5^	−0.10410	0.890396
Constant	0	5	0	10	0.632498	0.0134572	1.10 × 10^−5^	−0.10410	0.890396
Constant	0	5	0	5	0.632498	0.0134572	1.10 × 10^−5^	−0.10410	0.890396
Constant	0	5	0	10	0.632498	0.0134572	1.10 × 10^−5^	−0.10410	0.890396
Sinusoidal	5	2.5	0.25	10	0.626517	0.00597624	7.00 × 10^−6^	−0.10410	0.890396
Sinusoidal	5	2.5	0.25	10	0.626517	0.00597624	7.00 × 10^−6^	−0.10410	0.890396
Sinusoidal	5	2.5	0.25	5	0.626517	0.00597624	7.00 × 10^−6^	−0.10410	0.890396

## Data Availability

The data presented in this study are available on request from the corresponding author.
